# Fiber-Based Ultra-High-Speed Diffuse Speckle Contrast Analysis System for Deep Blood Flow Sensing Using a Large SPAD Camera

**DOI:** 10.3390/bios15080514

**Published:** 2025-08-07

**Authors:** Quan Wang, Renzhe Bi, Songhua Zheng, Ahmet T. Erdogan, Yi Qi, Chenxu Li, Yuanyuan Hua, Mingliang Pan, Yining Wang, Neil Finlayson, Malini Olivo, Robert K. Henderson, David Day-Uei Li

**Affiliations:** 1 Department of Biomedical Engineering, Faculty of Engineering, University of Strathclyde, Glasgow G4 0NW, UK; quan.wang.100@strath.ac.uk (Q.W.); chenxu.li@strath.ac.uk (C.L.); mingliang.pan@strath.ac.uk (M.P.); 2 A*STAR Skin Research Labs (A*SRL), Agency for Science, Technology and Research (A*STAR), 31 Biopolis Way, Nanos, Singapore 138669, Singapore; bi_renzhe@asrl.a-star.edu.sg (R.B.); zheng_songhua@asrl.a-star.edu.sg (S.Z.); qi_yi@asrl.a-star.edu.sg (Y.Q.); malini_olivo@asrl.a-star.edu.sg (M.O.); 3 School of Engineering, Integrated Nano and Micro Systems (IMNS), The University of Edinburgh, Edinburgh EH9 3JL, UK; ahmet.erdogan@ed.ac.uk (A.T.E.); yhua@exseed.ed.ac.uk (Y.H.); y.wang-410@sms.ed.ac.uk (Y.W.); n.finlayson@singularphotonics.com (N.F.); robert.henderson@ed.ac.uk (R.K.H.)

**Keywords:** DSCA, SPAD, blood flow, laser speckle

## Abstract

Diffuse speckle contrast analysis (DSCA), also called speckle contrast optical spectroscopy (SCOS), has emerged as a groundbreaking optical imaging technique for tracking dynamic biological processes, including blood flow and tissue perfusion. Recent advancements in single-photon avalanche diode (SPAD) cameras have unlocked exceptional sensitivity, time resolution, and high frame-rate imaging capabilities. Despite this, the application of large-format SPAD arrays in speckle contrast analysis is still relatively uncommon. This study introduces a pioneering use of a large-format SPAD camera for DSCA. By harnessing the camera’s high temporal resolution and photon-detection efficiency, we significantly enhance the accuracy and robustness of speckle contrast measurements. Our experimental results demonstrate the system’s remarkable ability to capture rapid temporal variations over a broad field of view, enabling detailed spatiotemporal analysis. Through simulations, phantom experiments, and in vivo studies, we validated the proposed approach’s potential for cerebral blood flow and functional tissue monitoring. This work highlights the transformative impact of large SPAD cameras on DSCA, setting the stage for breakthroughs in optical imaging.

## 1. Introduction

In a healthy individual, proper blood flow (BF) is vital for maintaining a steady supply of oxygen and essential energy sources, such as glucose and lactate, to organs, while also ensuring the efficient removal of metabolic waste products [1]. Of particular importance is cerebral blood flow (CBF), which is crucial for optimal brain function [2], brain metabolism [3], and the brain’s ability to respond to external stimuli [4]. For adults, typical CBF is around 50 mL/(100 g min) [5], whereas in newborns, it ranges between 10 and 30 mL/(100 g min) [6]. Any disruption to CBF can result in significant brain damage, including ischemic injury or stroke. Real-time monitoring of blood flow is essential for diagnosing and managing a variety of medical conditions, such as stroke [7], traumatic or hypoxic–ischemic encephalopathy (HIE) [8], neurological disorders, cardio-cerebral diseases, cancer treatments, tissue perfusion in peripheral vascular diseases [9], brain health and function [10], wound healing, sepsis, shock [11], skeletal muscle injuries [12], and tissue viability during surgical procedures.

Several optical methods have been employed for non-invasive monitoring of both healthy and pathophysiological tissues, including laser speckle contrast imaging (LSCI) [13], laser Doppler flowmetry (LDF) [14], diffuse correlation spectroscopy (DCS) [15], and diffuse speckle contrast analysis (DSCA) [16]—also referred to as speckle visibility spectroscopy (SVS) [17] or speckle contrast optical spectroscopy (SCOS) [18]. A key distinction between these techniques lies in their penetration depth. LSCI and LDF are typically limited to superficial tissues (less than 1 mm) due to their reliance on single or few dynamic scattering events, whereas DSCA/SCOS and DCS can penetrate much deeper, reaching several centimeters into tissue.

DCS relies on temporal sampling methods, where traditional avalanche photodiodes (APDs) or advanced single-photon avalanche diode (SPAD) detectors capture intensity fluctuations from one or a few speckle grains to reconstruct temporal dynamics. In contrast, DSCA uses a spatial sampling approach, which does not require a detector with a high frame rate. Instead, the camera typically operates with an exposure time longer than the speckle field’s decorrelation time, utilizing a larger detection area with many pixels to capture more photons and speckles. Originally, Bi et al. first proposed DSCA [16], drawing from LSCI, focusing on average values rather than imaging blood flow. DSCA is sometimes referred to as SVS [17] or SCOS [19], and it has been extensively studied theoretically [20,21] and experimentally [22,23]. Notably, Kim et al. [24] demonstrated that SCOS outperforms DCS, offering more than a 10-fold improvement in the signal-to-noise ratio (SNR) at a comparable cost. Importantly, while the setup for a fiber-based DSCA system is identical to that of DCS, DSCA does not necessarily require model-based fitting to analyze dynamic signals. However, quantitative blood flow estimation can still be achieved by analyzing speckle contrast, which, under sufficiently long exposure times, is inversely proportional to blood flow [25]. Conventionally, DSCA combines DCS’s deep tissue penetration capabilities with the relatively low-cost CCD or CMOS detectors used in LSCI. However, despite the use of these affordable detectors, DSCA has not yet seen widespread commercial adoption. With the introduction of faster photon-counting detectors, such as SPAD cameras, which have negligible readout noise, it is now possible to apply the spatial DSCA technique with improved statistical reliability in speckle contrast calculations.

Several similar speckle-based techniques have been developed to further advance BF imaging. For instance, speckle contrast diffuse correlation tomography (scDCT) [26] extends DSCA into three dimensions through tomographic reconstruction, enabling volumetric mapping of deep flow at the expense of increased complexity due to the need to scan the illumination using a galvo and the increased computational burden. Diffuse speckle contrast flowmetry (DSCF) [27] offers a wearable, fiber-free alternative that improves comfort and portability, particularly in neonates. Still, its penetration depth is limited to ~8 mm. Time-resolved LSCI (TR-LSCI) [28] enhances depth specificity by gating late-arriving photons via pulsed laser illumination and single-photon cameras, but limits depth sensitivity and spatial-temporal resolution. These techniques reflect a trade-off between depth, speed, spatial resolution, and implementation complexity.

This study introduces a novel, compact 512 × 512 pixels SPAD array called ATLAS [29,30] built with industry-standard CMOS technology, for deep tissue BF measurements using the DSCA approach. This detector array boasts a high photon efficiency, minimal dead time, zero readout noise, and a high frame rate (up to 27 kfps), all essential for precise BF measurements in low-light, in vivo conditions. Future advancements could enable real-time BF monitoring in freely moving subjects. To evaluate our ATLAS-DSCA system, we measured deep tissue BF in four healthy volunteers during arterial arm cuff occlusion and forehead BF monitoring. Additionally, vibration phantom experiments were performed, and the results were compared to those from a traditional DSCA system.

A summary of existing DSCA/SCOS systems is provided in Table 1, detailing laser wavelength, applications, sampling rate, source-detector separation, and sensor type. This table highlights the evolution of DSCA technology and contextualizes the significance of our proposed system in advancing the field.

The structure of this paper is as follows: we first present the theoretical principles of DSCA, followed by a description of the SPAD camera architecture and system implementation. Next, we compare our phantom results with those from traditional DSCA methods. Finally, we present the in vivo experimental results, discuss the advantages and limitations of the SPAD-based DSCA system, and propose potential future improvements.

## 2. Materials and Methods

### 2.1. Theoretical Background

DSCA relies on the speckle contrast (κ), defined as the ratio of the standard deviation ($\sigma_{I}(\rho ,T)$) of the measured intensity during a specific exposure time to its mean ($\langle I\rangle$) across various speckles. DSCA quantifies the statistics of the fluctuation of the speckle pattern as the variance of the measured intensity ($\sigma_{I}^{2}$) either in spatial or temporal domains [38]

(1) $$\kappa^{2}\left(\rho ,T\right)=\frac{\sigma_{I}^{2}(\rho ,T)}{\langle I(\rho ,T)\rangle^{2}}$$

where ρ denotes the source-detector separation, *T* is the exposure time, and $\kappa^{2}$ varies between zero and one, with a higher value indicating a slower scatterer fluctuation. *κ* is related to the normalized electric field auto-correlation function ($g_{1}(r,T)$) and is given by:

(2) $$\kappa^{2}\left(\rho ,T\right)=\frac{2\beta }{T}\int_{0}^{T}\left(1-\frac{\tau }{T}\right){\left|g_{1}\left(\rho ,\tau \right)\right|}^{2}d\tau .$$

where $g_{1}\left(\rho ,\tau \right)=G_{1}(\rho ,\tau )/G_{1}(\rho ,0)$ and $G_{1}(\rho ,\tau )$ represents the Green’s function of Brownian motion at ρ in a semi-infinite geometry [39]. In this equation, β is an experimental constant that accounts for the collection optics.

### 2.2. Noise Correction

In practical applications, it is crucial to adjust the speckle contrast calculation to account for shot noise and other noise sources inherent in the detection system. Since speckle contrast (κ) is influenced by the variance from the expected theoretical behavior, deviations become particularly pronounced in regions with a lower signal-to-noise ratio (SNR). It is important to note that the calculation in Equation (1) does not account for these additional noise contributions, especially in areas with a lower SNR. To address this, we define a corrected squared speckle contrast $\kappa_{c}^{2}$ as follows [40]:

(3) $$\kappa_{c}^{2}=\kappa_{m}^{2}-\kappa_{s}^{2}-\kappa_{d}^{2}-\kappa_{r}^{2}-\kappa_{q}^{2}$$

where

$\kappa_{m}$: the measured contrast computed from camera images (after any dark offset subtraction);
$\kappa_{s}$: the contrast arising from photon shot noise (Poisson statistics of photon arrival);
$\kappa_{r}$: the contrast contribution from camera read-out noise;
$\kappa_{d}$: dark current and other system noise induced contrast;
$\kappa_{q}$: the quantization-induced contrast.

Before calculating κ, the raw intensity images are corrected by subtracting dark counts and removing bad pixels. The dark offset, determined as the average of 1000 dark images (with the laser off), is subtracted from the raw intensity to obtain the corrected intensity $I_{c}=I-I_{D}$. Even though the intensity is corrected, the variance from the dark noise is included in the intensity’s variance, making it essential to deduct the dark variance $\sigma_{D}^{2}$. Another significant noise source is inherent shot noise, which follows Poisson statistics, with a variance equal to the mean intensity in electrons [e-], defined as $\sigma_{s}^{2}=\langle I_{c}\rangle$. Due to their single-photon detection mechanism with a massive conversion gain (the output is digital), SPAD arrays inherently avoid the analog readout and quantization errors typically encountered in CMOS/CCD-based systems [41], Equation (3) becomes:

(4) $$\kappa_{c}^{2}=\frac{\sigma_{I_{c}}^{2}-\sigma_{D}^{2}-\sigma_{s}^{2}}{{\langle I_{c}\rangle }^{2}}$$

The blood flow index (BFi) is then related to $\kappa_{c}^{2}$ by:

(5) $$BFi=\frac{1}{\kappa_{c}^{2}}$$

In all the findings presented in this paper, we use the normalized blood flow index (normalized BFi) to provide standardized BF data, enhancing comparability across measurements. Similar to DCS, BFi does not directly quantify bulk volumetric blood flow but rather reflects the dynamics of particle displacement, which is more accurately characterized by a shear-induced diffusion process modeled as a random walk [15]. As such, BFi is particularly sensitive to microvascular flow characteristics, including variations in perfusion, vessel architecture, and flow heterogeneity.

### 2.3. SPAD (ATLAS) Architecture

The sensor, called ATLAS hereafter, features a 512 × 512 array of deep trench isolation (DTI) microlensed SPADs with a 10.17 µm pitch. It offers a peak photon detection efficiency (PDE) of 55% (26% at 940 nm) and a median dark count rate (DCR) of 500 cps at room temperature, operating at 23 V with a breakdown voltage of 17.8 V [42]. The passively quenched SPADs are organized into 4 × 4 groups using an OR tree structure to form 128 × 128 macropixels, each with a 40.68 µm pitch. Each macropixel can compute a 31-tap autocorrelation function with a minimum correlation time of 1 µs (which can be reduced to 100 ns, depending on the clock rate). ATLAS supports the following operating modes:

1. The 22-bit single-photon counting mode;

2. The time-gated 22-bit single-photon counting mode via a balanced H-tree, with time gates generated by an FPGA or on-chip DLL at 15–30 ps granularity;

3. The multispeckle DCS mode, delivering the average of individual pixel autocorrelations in an on-chip, high-speed “ensemble” mode [29,30];

4. The DCS imaging mode [29,30].

In ATLAS, each photon is directly converted into a 1-bit count, eliminating the readout noise typically encountered in CCD or CMOS imagers. The clock frequency is adjustable (e.g., 20 MHz, 25 MHz, 50 MHz, 75 MHz), and the parameter TINT_TBIN_ITERATIONS defines the number of on-chip accumulations performed before data readout, which are used to control the exposure time via our custom software. The TINT_TBIN_ITERATIONS can range from 1 to 65,535. The chip is mounted on a PCB board and connected to a field-programmable gate array (FPGA), such as the Opal Kelly 7310-A200.

**Figure 1:**
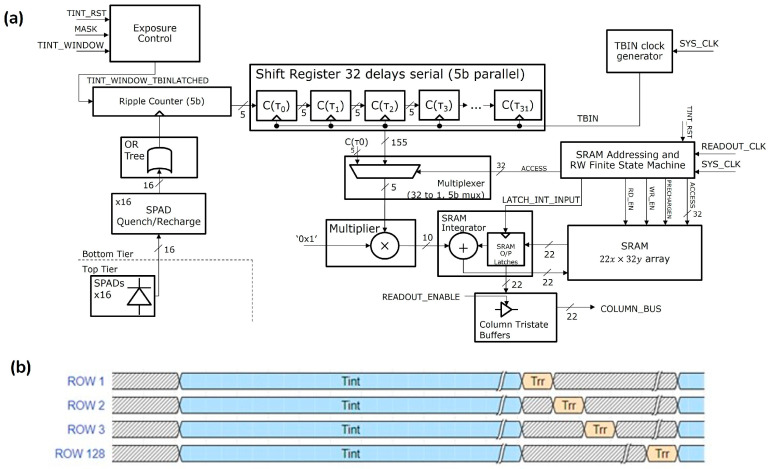
(**a**) Block diagram showing main blocks of the pixel schematic operating in photon counting mode. (**b**) Global shutter timing diagram for photon counting mode. Integration time: Tint; Row readout time: Trr.

In the photon counting (PC) mode, as shown in Figure 1a, each pixel acquires photons during a single exposure (TBIN period), and photons are counted with a 5-bit ripple counter. At the end of the TBIN exposure, the shift register is clocked, transferring the 5-bit count into the first element (C(τ_0_)) which is subsequently accumulated at the first SRAM address. This is repeated for a user-defined number of integration cycles (i.e., TINT_TBIN_ITERATIONS). After this, the accumulated 22-bit count is read out via the column bus. It is worth noting that in the PC mode, only the first elements of the Shift Register and SRAM are utilized, and the Multiplier is configured to multiply C(τ_0_) by 1 (effectively by-passing the Multiplier). The remaining 31 shift register and SRAM elements are utilized in other DCS-related modes. Figure 1b shows a Global shutter timing diagram for the photon counting mode.

### 2.4. Implementation (Experimental Setup)

**Figure 2:**
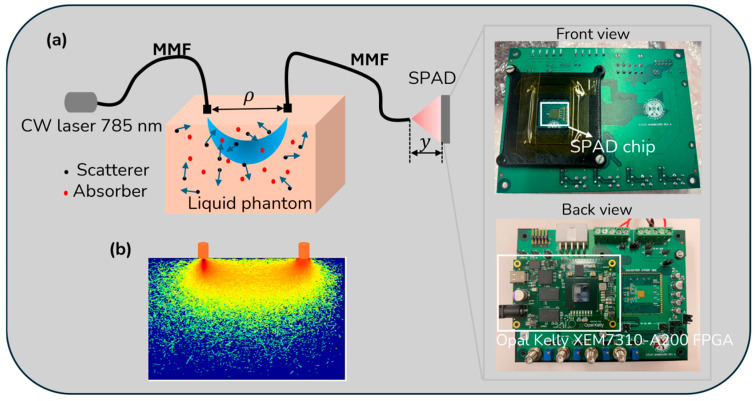
(**a**) The schematic of the experimental setup illustrates the optical measurement system using multimode fibers (MMFs) and a single-photon avalanche diode (SPAD). A continuous-wave (CW) laser (785 nm) propagates through a liquid phantom containing scatterers and absorbers, represented by black and red dots, respectively. y indicates the vertical position of the SPAD detector. Zoomed-in images show the SPAD chip (front view) and the Opal Kelly FPGA board (back view). (**b**) Simulation results depict scattered light traveling through a “banana-shaped” region in tissue. These simulations were conducted using MCmatlab [43] based on the model MCXYZ developed by Jacques and Li [44]. Detailed simulation settings can be found in Ref. [45].

The experimental setup is schematically illustrated in Figure 2a. A long-coherence 785 nm laser (>5 m coherence length, DL785-100-S from CrystaLaser, Reno, NV, USA) is the light source, and the PDE is 47% at 785 nm. Laser light is delivered to the phantom through a multimode (MM) fiber with a 200 µm core, while scattered light is collected by another MM fiber with the same core size. One end of the detection fiber is placed on the phantom’s surface, with the other end aligned to the center of the SPAD camera. To attenuate the laser beam output to 30 mW, which meets ANRS safety requirements, a variable neutral density filter (NE205B, OD = 0.5, Thorlabs, Cambridgeshire, UK) was placed between the laser and the source fiber tip. We carefully adjusted the distance between the detection fiber tip and the sensor surface (y) to reduce spatial heterogeneity. During this process, we monitored the speckle pattern in real time and fine-tuned the distance until the spatial intensity distribution appeared uniform across the sensor. After confirming the absence of significant spatial heterogeneity, we acquired and used the data for final analysis. Zoomed-in images of the SPAD chip (front view) and the Opal Kelly FPGA board (back view) are provided.

Figure 2b displays simulation results of scattered light propagation through a “banana-shaped” region in tissue, simulated using MCmatlab [43] based on the MCXYZ model developed by Jacques and Li [44]. The simulation settings are detailed in Ref. [45]. Considering the SNR and flow sensitivity for speckle imaging in biomedical applications, typical exposure times range from 1 to 10 ms [46]. In our experiments, we varied the exposure time by adjusting TINT_TBIN_ITERATIONS. For instance, at an exposure time of 1.64 ms (TINT_TBIN_ITERATIONS = 1024) with ρ = 15 mm, the system achieved an image acquisition rate of 361.7 fps (= 1/(the exposure time + the frame readout time)).

### 2.5. Data Processing

**Figure 3:**
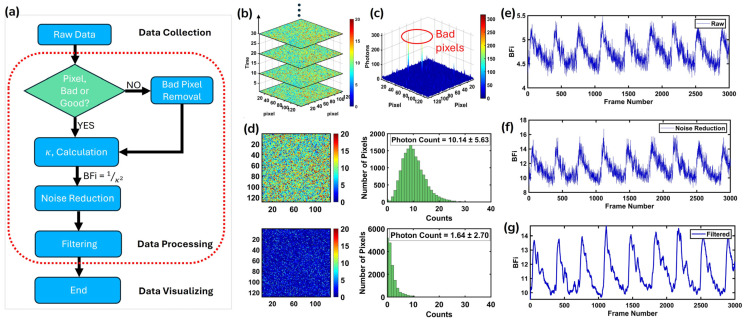
(**a**) The DSCA data analysis workflow, including data collection, processing, and visualizing. (**b**) The raw speckle data obtained from a human arm experiment at different time points, including the 1st, 10th, 20th, and 30th frames. (**c**) After acquiring the raw data, bad pixels (highlighted in red circles) were identified and removed, followed by κ calculation, noise reduction, and final filtering. (**d**) The speckle image and the corresponding photon histogram (photons: 10.3 ± 5.63) shown in the upper row of the panel (**d**), with the dark image taken under the same detector conditions and its corresponding photon histogram (photons: 1.99 ± 2.70) in the bottom row of the panel (**d**). (**e**), (**f**), and (**g**) stand for the BFi from raw data, after noise reduction and after filtering, respectively.

The data processing pipeline for speckle contrast analysis is illustrated in Figure 3 and follows a structured approach to ensure high-quality and reliable data. Raw speckle data, also known as intensity imaging data under PC mode, is initially acquired as a three-dimensional matrix. Once the data is acquired, a pixel quality assessment is performed to identify and eliminate bad pixels that introduce artifacts or bias into the analysis. Specifically, ‘hot’ pixels—those with significantly higher dark counts [29]—are detected and removed using a 3-sigma rule. Any pixel that deviates more than three standard deviations from the mean is replaced with NaN to prevent its influence on subsequent calculations, implemented with:

(6) $$I\left[\left|I-\bar{I}\right|>3\sigma \right]=NaN$$

where I is the frame data, $\bar{I}$ is the mean intensity of frame data, and σ is the standard deviation of the frame data. Once bad pixels are identified, they are excluded from further calculations. For valid pixels, the speckle contrast κ is calculated over the entire pixel array (128 × 128), rather than using a smaller local window (e.g., 3 × 3, 5 × 5, or 7 × 7) like previous studies [46], and BFi is subsequently derived based on BFi = 1/κ^2^. The resulting signal is then subjected to noise reduction algorithms to minimize fluctuations caused by external disturbances or system imperfections. Filtering techniques, including a smoothing window of 20, are applied to refine the data and highlight physiologically relevant blood flow variations. Finally, the processed data is visualized, enabling the interpretation of BFi dynamics over time. This pipeline ensures the extraction of accurate and meaningful blood flow information while minimizing the impact of noise and artifacts.

## 3. Results

### 3.1. Simulation Results

**Figure 4:**
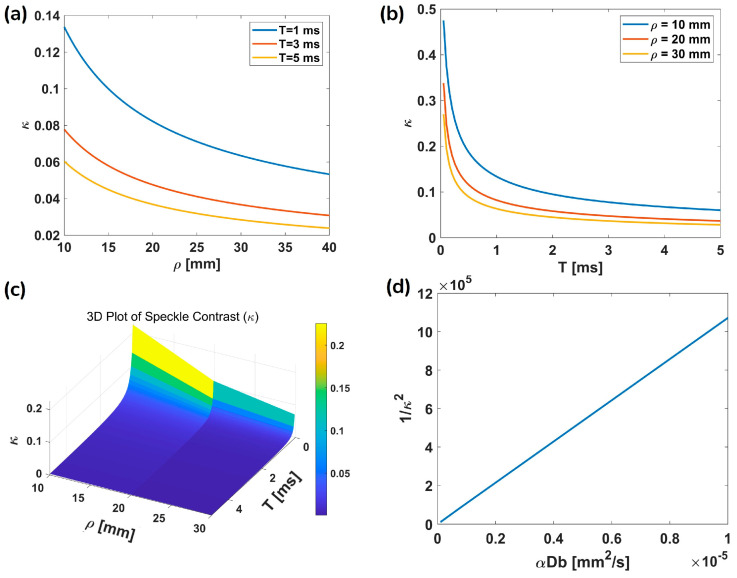
Numerical simulations showing the relationship between κ and various parameters. (**a**) κ as a function of ρ for T=1,3,5 ms. (**b**) κ as a function of T for ρ=10,20,30 mm. (**c**) 3D visualization of *κ* as a function of both ρ and *T*. (**d**) A linear relationship between $1/\kappa^{2}$ and $\alpha D_{b}$, showing the expected theoretical trend, with T = 2 ms. Here λ=785 nm, $\mu_{a}=0.01\;{\mathrm{mm}}^{-1}$, $\mu_{s}^{\prime }=1\;{\mathrm{mm}}^{-1}$, and n=1.33.

As a function of ρ and T, κ is computed using Equation (2) and presented in Figure 4a,b. In Figure 4a, κ decreases with increasing ρ for T = 1, 3, and 5 ms with a smaller T exhibiting a higher κ. This trend aligns with the expectations that a larger ρ leads to greater photon scattering and diffusion, thereby reducing κ. Figure 4b demonstrates the nonlinear decrease in κ and T at various ρ values. As T increases, κ decreases in a nonlinear fashion, with the decrease being more pronounced at a smaller ρ, indicating that a shorter T captures more high-frequency speckle fluctuations associated with BF. To further explore this dependency, Figure 4c presents a 3D visualization of κ as a function of ρ and T. The surface plot clearly shows that *κ* is highest at a smaller *ρ* and *T*, confirming the interplay between spatial and temporal factors in speckle contrast dynamics. Finally, Figure 4d presents the relationship between 1κ2 and $\alpha D_{b}$ (BFi), where it is evident that BFi increases linearly with 1κ2. The simulations were conducted with λ = 785 nm, $\mu_{a}$ = 0.01 mm^−1^, $\mu_{s}^{\prime }$ = 1 mm^−1^, and the refractive index n = 1.33, representing a typical tissue-mimicking medium. The parameters are comprehensively integrated into Equation (A1), with the full implementation steps outlined in Appendix A.

### 3.2. Measurement Flexibility

The measurement flexibility of the ATLAS-DSCA was assessed by varying the TINT_TBIN_ITERATIONS parameter in our custom software. Table 2 summarizes the relationship between TINT_TBIN_ITERATIONS, exposure time, frame readout time, frame time, and frame rate, for some example TINT_TBIN_ITERATIONS, assuming global shutter mode, a 20 MHz (equivalent period of 50 ns = 1/20 MHz) clock frequency, and TBIN_CLK_PERIODS = 32, where the TBIN_CLK_PERIODS define the minimum exposure time. As the exposure time increases, the frame rate decreases, highlighting the impact of a longer integration period on the data acquisition speed. Specifically, increasing TINT_TBIN_ITERATIONS leads to a proportional rise in the exposure time, significantly reducing the achievable frame rate. For instance, with a clock frequency of 20 MHz and TINT_TBIN_ITERATIONS set to 32, the system achieves a frame rate of 849.2 fps. However, at TINT_TBIN_ITERATIONS = 65,535, the frame rate drops to 9.4 fps.

**Figure 5:**
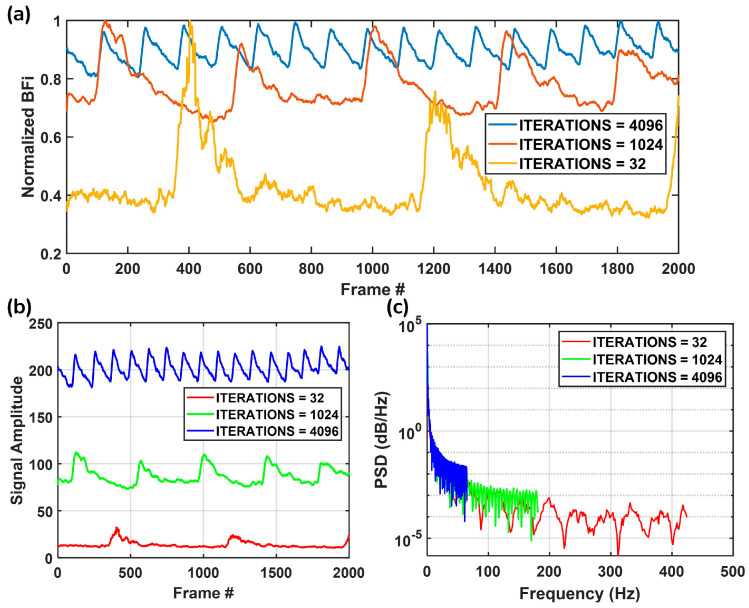
(**a**) Normalized BFi measured over time with varying TINT_TBIN_ITERATIONS values (32, 1024, and 4096). (**b**) Raw signal amplitudes across different iteration settings, highlighting variations in signal intensity and noise levels. (**c**) The power spectral density (PSD) analysis of the signals, demonstrating the impact of iteration settings on frequency content, where lower iterations allow for higher frequency resolution whereas higher iterations result in smoother signals with reduced high-frequency components.

Figure 5 illustrates the impact of different TINT_TBIN_ITERATIONS values (32, 1024, and 4096) on normalized BFi measurements over the frame number. The measurements were conducted on the left arm of a healthy volunteer at ρ = 12 mm (laser power: 4 mW), with the SPAD sensor set to a clock frequency of 20 MHz and TBIN_CLK_PERIODS = 32. The clock frequency is also adjustable (e.g., 50 MHz, 75 MHz) depending on experimental requirements. Each TINT_TBIN_ITERATIONS value corresponds to a different frame rate, as shown in Table 2, which represents the sampling rate. For example, TINT_TBIN_ITERATIONS values of 32, 1024, and 4096 correspond to sampling rates of 849.2 Hz, 361.7 Hz, and 130.2 Hz, respectively. Fewer iterations result in a higher temporal resolution, allowing for the capture of rapid dynamics. In contrast, more iterations produce smoother curves due to the enhanced SNR (SNR = $20{log}_{10}(S/N)$, where S and N represent the signal and noise counts when the sensor is an SPAD), albeit with a reduced temporal resolution. Here, we obtain SNR values of 12.10 dB, 25.13 dB, and 30.32 dB for TINT_TBIN_ITERATIONS = 32, 1024, and 4096, respectively. A lower TINT_TBIN_ITERATIONS means a shorter *T*, leading to a lower κ, which agrees with the simulation results shown in Figure 4b.

Figure 5b presents the raw signal amplitude for the three iteration settings, highlighting the increased signal intensity and noise levels associated with lower iterations. In Figure 5c, the power spectral density (PSD) analysis, computed using Welch’s method, further reveals the impact of iteration settings on signal frequency content. Welch’s method can estimate the PSD by segmenting the signal into overlapping sections, applying a window function, and averaging the periodograms. The PSD estimate using Welch’s method is given by:

(7) $$\;PSD\left(f\right)=\frac{1}{M}\sum_{m=1}^{M}P_{m}\left(f\right),P_{m}\left(f\right)=\frac{1}{N}{\left|FFT(x_{m})\right|}^{2},$$

where *f* is the frequency at which the PSD is estimated, M is the number of overlapping segments into which the signal is divided, and $P_{m}(f)$ is the periodogram (power spectrum for a given segment) of the *m-th* segment at f. *N* is the length of each segment, and $x_{m}$ is the m-th segment of the signal. FFT stands for Fast Fourier Transform. By averaging multiple periodograms, Welch’s method reduces variance and provides a smoother representation of the frequency components. The low iteration setting (red) allows for higher-frequency components to be preserved. In contrast, increasing the iterations results in a more attenuated high-frequency response, favoring smoother signals. These findings highlight the inherent trade-off between temporal resolution and signal quality: fewer iterations improve temporal tracking but introduce higher noise, whereas more iterations enhance signal stability at the cost of high-frequency information. This balance underscores the system’s versatility in adapting to diverse experimental requirements.

### 3.3. Phantom and In Vivo Measurements

#### 3.3.1. Phantom Measurements

To validate the ATLAS-DSCA system, phantom experiments at ρ= 12 mm were conducted using a configuration that allowed direct comparison with the conventional CMOS-DSCA system, which has been well established by A*STAR and employs a Sony IMX249 CMOS sensor (MER2-231-41U3M). A custom-made solid silica phantom was embedded with a vibration motor whose intensity could be externally controlled to simulate dynamic scattering. Since the aim was to qualitatively assess the system’s response to flow-like changes rather than to obtain absolute flow values, the optical properties of the phantom were not measured. This design allowed us to focus on the system’s sensitivity and robustness in detecting relative changes in speckle contrast.

**Figure 6:**
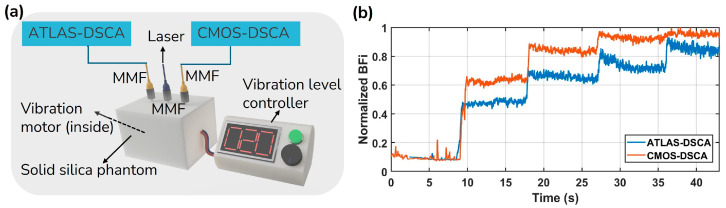
(**a**) Experimental setup for DSCA: A laser illuminates a solid silica phantom via an MMF, with scattered light collected by two separate MMFs for ATLAS- and CMOS-DSCA detection. A vibration motor inside the phantom generates controlled motion at intensities 0, 12, 24, 36, 52. (**b**) Normalized BFi over time for ATLAS-DSCA (blue) and CMOS-DSCA (orange), showing responses to varying vibration levels.

Figure 6a shows the experimental setup, where a laser illuminates the solid silica phantom through an MMF, and scattered light is collected via two separate MMFs: one connected to the ATLAS-DSCA system and the other to the CMOS-DSCA system. A vibration motor embedded in the phantom induces controlled motion, with vibration levels (0, 12, 24, 36, 52) adjusted through an external controller. Figure 6b presents the time-dependent normalized BFi measured by both systems. The stepwise increases in BFi correspond to different vibration levels applied to the phantom, illustrating the responsiveness of ATLAS-DSCA and CMOS-DSCA to dynamic changes. A noticeable discrepancy between the BFi values obtained from the two systems likely reflects inherent differences in their measurement characteristics, such as quantum efficiency, dark current, and other detector-specific factors, between CMOS and SPAD detectors. This discrepancy validates the feasibility of the ATLAS-DSCA method under controlled conditions, paving the way for its application in complex in vivo scenarios.

#### 3.3.2. Arm Cuff Occlusion

**Figure 7:**
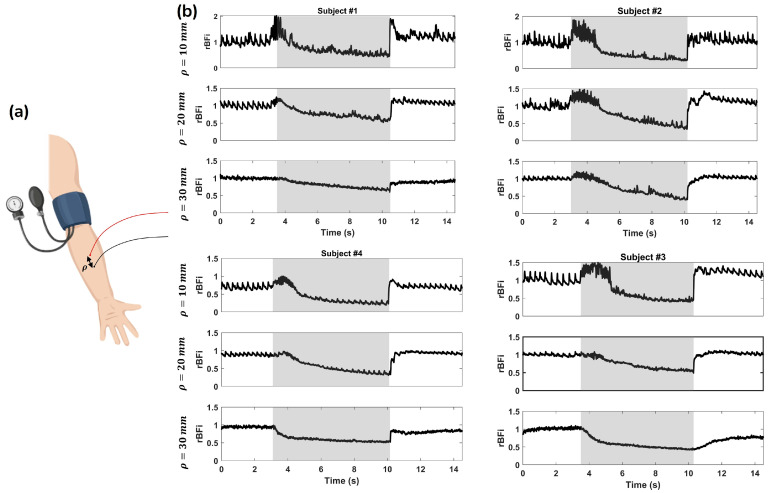
(**a**) Schematic representation of the experimental setup, where a blood pressure cuff is placed on the upper arm to induce controlled vascular occlusion, with a certain ρ. (**b**) Temporal rBFi variations at ρ = 10, 20, and 30 mm for four subjects (Subjects #1–#4). The gray-shaded regions indicate the period of cuff inflation, during which blood flow is restricted. The signals show a characteristic decrease in rBFi during occlusion, followed by a recovery phase after cuff deflation.

To further demonstrate the ATLAS-DSCA system, in vivo measurements were conducted using an arm cuff occlusion model at ρ = 10, 20, and 30 mm. As shown in Figure 7a, volunteers sat comfortably with their left arm placed on a pad and the ATLAS-DSCA probe attached to the wrist. Figure 7b presents relative BFi (rBFi = BFi/BFi_baseline_) time series data for four subjects at different ρ values, highlighting the microvascular hemodynamic changes during occlusion and subsequent recovery. After a baseline period of approximately 3.5 s, a blood pressure cuff was inflated to 200 mmHg for 7 s. Upon cuff release, blood flow recovery was recorded for 4 s. The gray-shaded regions indicate the cuff inflation period, during which a marked decrease in rBFi is observed. Upon cuff release, the rBFi overshoots to a hyperemic value much larger than the baseline, as expected, with varying recovery kinetics across subjects and measurement depths. Notably, the reduction in rBFi is more pronounced at a shorter *ρ* (*ρ* = 10 mm), suggesting that superficial microvascular networks exhibit a stronger response than deeper tissues, where the SNR may be lower. To mitigate this issue, one practical solution is to use a larger diameter detection fiber (e.g., 1000 µm) in place of the 200 µm fiber currently used. This would increase photon collection efficiency and improve SNR, particularly under low-perfusion conditions. Table 3 presents the SNR values, dark count levels, and average intensity counts of the raw data for a representative subject under different experimental conditions.

#### 3.3.3. In Vivo Forehead Measurements

**Figure 8:**
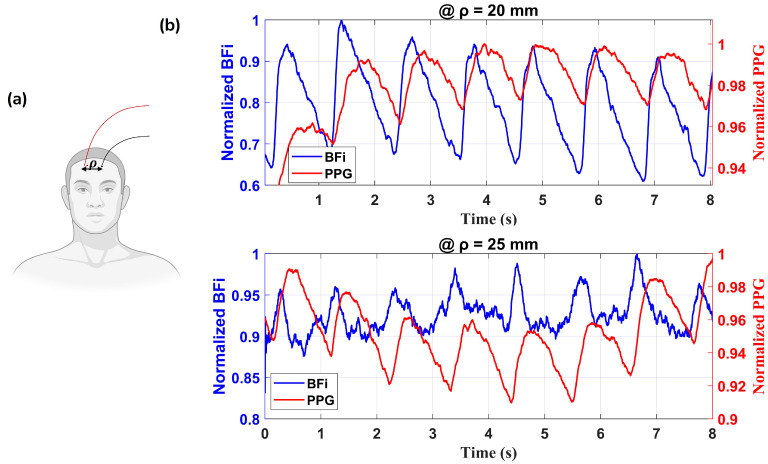
(**a**) Schematic representation of the measurement setup. (**b**) Normalized BFi (in blue) and photoplethysmography (PPG, in red) signals at ρ = 20 and 25 mm. The signals exhibit a clear correlation, reflecting hemodynamic fluctuations over time.

ATLAS-DSCA was also applied to monitor cerebral blood flow on the adult human forehead, as shown in Figure 8a. Figure 8b represents the temporal variations in normalized BFi (blue) and photoplethysmography (PPG, red) signals at *ρ* = 20 mm and *ρ* = 25 mm. The BFi reflects cerebral blood flow fluctuations, whereas the PPG indicates peripheral blood volume changes. A clear phase shift between the two signals is observed, highlighting the differences in hemodynamic responses at varying tissue depths. The average intensity, which is similar to the PPG signal, serves as an indicator of blood volume [47]. In our measurements, the BF waveform exhibits sharper peaks and more detailed features within each cardiac cycle than the PPG waveform, with the BF peak consistently preceding the blood volume peak—a phenomenon also reported in Ref. [32].

**Figure 9:**
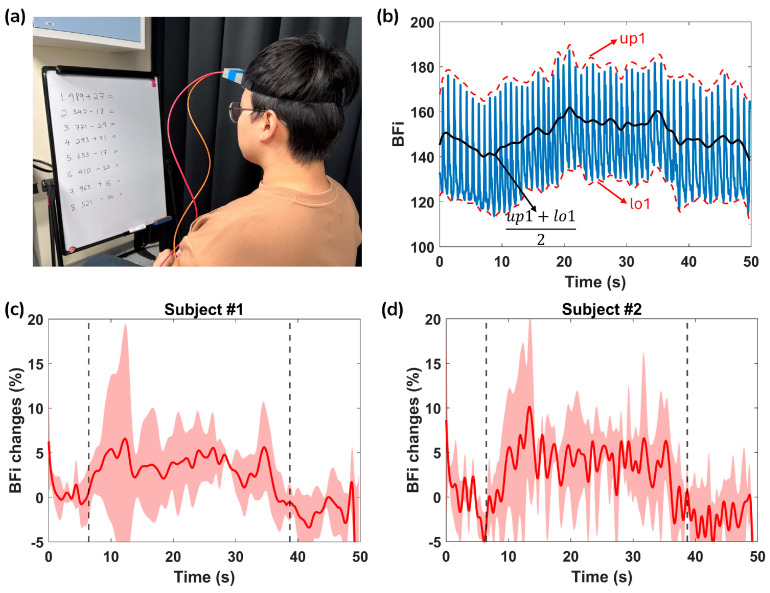
(**a**) The experimental setup showing a subject performing mental arithmetic tasks while cerebral blood flow was monitored using a home-made head-mounted optical probe. (**b**) The recorded BFi (in blue) during the task. The red dotted lines represent the upper and lower envelopes (up1 and lo1, respectively), computed using the MATLAB envelop function with the ‘peak’ method. The black line represents the average of up1 and lo1, providing a smoothed trend of BFi signal. (**c**) and (**d**) Relative changes in BFi (%) for Subject #1 and Subject #2, respectively. The shaded region represents the standard deviation, whereas the dashed vertical lines indicate the start and end of the cognitive task.

To further demonstrate the sensitivity of ATLAS-DSCA, Figure 9 presents cerebral function monitoring during a mental arithmetic task (at *ρ* = 20 mm) in two subjects. Figure 9a shows the experimental setup, where the probe was placed on the forehead, targeting the prefrontal cortex, which plays a key role in cognitive processes such as reading unfamiliar text, planning, and working memory [48,49]. In this experiment, subjects rested for 8 s before being presented with math questions for 30 s, followed by a recovery period after removing the questions. Figure 9b illustrates a representative BFi measurement from one subject. The upper and lower envelopes of the BFi signal (denoted as up1 and lo1, respectively) were calculated using MATLAB(2023b)’s envelope function with the ‘peak’ method (window size = 250). The trend in the BFi signal is defined as:

(8) $${BFi}_{signal}=\frac{up1+lo1}{2}$$

Taking the first 8 s as the baseline, the percentage change in BFi is calculated as:

(9) $$\mathrm{BFi}\;\mathrm{changes}=\frac{\left|{BFi}_{signal}-{BFi}_{baseline}\right|}{{BFi}_{baseline}}\times 100\%$$

As shown in Figure 9c,d, we observed that blood flow significantly increased by 3.8% to 10.1% (average over three trials per subject) during activation and returned to baseline values post-activation, consistent with expected physiological responses. The shaded area represents the standard deviation of the three trials for each subject, calculated using built-in MATLAB functions. These findings demonstrate the feasibility of using ATLAS-DSCA to track neurovascular dynamics in response to cognitive stimuli, offering valuable insights into cerebral hemodynamic responses associated with mental effort.

## 4. Discussion

The proposed ATLAS-DSCA system leverages a 512 × 512 SPAD array for non-invasive deep tissue blood flow measurement. Our findings show that the ATLAS-DSCA offers a comparable performance to traditional CMOS-DSCA systems but with the added benefit of a higher sampling rate. Validation studies, including arm cuff occlusion, in vivo forehead measurements, and cognitive task trials in healthy subjects, all confirm the robustness and effectiveness of our approach.

Phantom experiments (Figure 6b) conducted alongside a CMOS-DSCA system further validate the reliability of the ATLAS-DSCA. Additionally, arm cuff occlusion studies (Figure 7b) highlight the system’s ability to assess deep tissue blood flow, demonstrated by the pronounced hyperemic response following cuff release. Cognitive task experiments (Figure 9c,d) also show a marked increase in blood flow during mental arithmetic, aligning with previous research [24,50].

As presented in Table 1, Kim et al.’s SCOS system achieved ρ = 40 mm with a sampling rate of 160 Hz [24]. However, their approach involves inevitable trade-offs, such as using lower-cost CMOS cameras, which introduce higher readout noise, reduced bit depth, and nonlinear or non-uniform camera gains. To improve photon flux without exceeding safety limits, they implemented a pulsing strategy (10% duty cycle) with an optical chopper on a volume holographic laser, resulting in a more complex optical setup. Additionally, their system incorporates a complicated 4f structure, making it bulkier and less portable.

In contrast, Huang et al.’s system [34] achieved ρ = 50 mm with a sampling rate of 80 Hz, which captures low-frequency signals (<65 Hz), unable to resolve high-frequency components, as shown in Figure 5c (blue curve), which may result in missing critical information. However, our ATLAS-DSCA system can resolve high-frequency signals (>200 Hz), making it ideal for analyzing dynamic, rapidly changing physiological signals. They used a higher laser power (45 mW) to achieve ρ = 50 mm. Their detection probe is designed to place CMOS image sensors close to the patient’s skin, eliminating the need for detection fibers. This increases photon collection and improves the SNR, making it ideal for adult stroke diagnostics. However, this design is less suitable for premature or neonatal patients, requiring minimal disruption.

In comparison, our ATLAS-DSCA system, while achieving ρ = 25 mm, distinguishes itself with an exceptional sampling rate exceeding 800 Hz. It can detect human blood flow, and is also suitable for monitoring animal health and wellbeing (for small animals, the heart rate typically ranges from 4 to 12.5 Hz, or 240 to 750 beats per minute [51]). This high sampling rate also opens the door to broader applications, including ultrasound detection, assuming the SPAD’s bandwidth and sensitivity support higher frequencies. Our system’s flexibility is a significant advantage: the ATLAS-DSCA system can operate in the DCS mode, providing ρ = 50 mm (to be published separately) using on-chip autocorrelators [29,30]. Several studies [18,27,52,53] have compared DCSA/SCOS and DCS technologies, employing either Monte Carlo simulations or separate detectors—one dedicated to DCSA/SCOS and another to DCS. In contrast, our DSCA/DCS system allows for switching between DCS and DSCA modes, facilitating a more controlled and direct comparison.

Furthermore, there are two main approaches for capturing temporal dynamics: temporal sampling methods (such as DCS using SPAD arrays) and speckle ensemble methods (like SVS/DSCA and LSCI). While temporal sampling methods require extremely high frame rates (above 26 kfps, as highlighted in Wang et al.’s review [39]) to capture fast fluctuations, speckle ensemble methods improve SNR by integrating over multiple speckles. Our work demonstrates that, although SPAD sensors may not be ideal for traditional DCS (calculating $g_{2}$ using sequential frame data), they hold significant promise in DSCA, SCOS, and SVS applications.

It is important to note that our SPAD array maintains a high SNR even with very short exposure times (as low as 51.2 µs), thanks to its lack of readout noise and minimal dead time—critical advantages that enhance the performance of the ATLAS-DSCA system.

Also, it is worth noting that our SPAD camera can operate in time-gating mode, similar to the work of Fathi et al. [28], who utilized SwissSPAD2 (512 × 512) with a pulsed laser for laser speckle imaging. Time-gated detection can reduce partial volume effects and achieve a spatial resolution of 1–2 mm when imaging deep brain regions in adult rats. Therefore, comparing under the same time-gated mode in future studies would be valuable.

However, two main challenges have limited the widespread integration SPAD technology into DSCA/SCOS systems so far: (1) Manufacturing large-format SPAD arrays with uniform performance is challenging, and the readout of many high-speed channels is non-trivial. Thus, while SPAD arrays are growing in size, standard cameras still offer orders of magnitude more pixels for wide-field SCOS measurement; (2) SPAD-based imaging systems are not yet mass-produced; therefore, they are more expensive and less readily available than CMOS or CCD cameras. A particularly critical issue is the extremely high data throughput generated by large SPAD arrays, which can easily overwhelm existing readout and data transfer interfaces, causing latency in speckle contrast computation. Future improvements could include on-chip preprocessing and data compression to reduce raw data volume, and the adoption of high-bandwidth transfer links (e.g., PCIe Gen4 or optical interconnects) and GPU/FPGA-based real-time processing architectures. Additional advances in power efficiency, thermal management, and compact system integration will also be needed to make SPAD-DSCA technology more practical and deployable for bedside, real-time clinical monitoring.

Another limitation of this study is that the observed blood flow changes during mental arithmetic (ranging from 3.8% to 10.1%) are slightly lower than the typically reported values of approximately 7% to 16% [24]. This discrepancy is likely due to the absence of conventional neuroimaging tools—such as PET or fNIRS commonly used to accurately localize subject-specific activation regions. We acknowledge this limitation and plan to address it in future work by incorporating such tools to improve spatial localization.

One more limitation is that achieving ρ = 30 mm is challenging in our current setup due to the limited SNR. One potential solution in our future work to address this limitation is replacing the current 200 µm detection fiber with a larger-diameter fiber (e.g., 1000 µm), which could improve photon collection efficiency and enhance SNR.

Despite these challenges, we still believe that recent advancements in SPAD technology—such as the development of large-area SPAD arrays (512 × 512) and improved time-gated detection—suggest that SPADs could play a more prominent role in next-generation DSCA/SCOS applications. Their unparalleled temporal resolution, high sampling rate, and ability to perform time-gated measurements make them particularly attractive for deep-tissue blood flow imaging and functional neuroimaging applications.

## 5. Conclusions

In conclusion, we present a groundbreaking fiber-based, ultra-high-speed DSCA system using a large-format SPAD camera for non-invasive deep tissue blood flow sensing. By combining a custom-designed SPAD array with cutting-edge optical and signal processing techniques, our system achieves an exceptional temporal resolution and sensitivity, enabling the capture of rapid, dynamic blood flow variations across a broad field of view. Extensive experimental validations—from phantom studies and cuff occlusion tests to in vivo measurements—demonstrate that our DSCA system reliably detects deep tissue hemodynamic changes and outperforms traditional CMOS-based systems in terms of sampling rate. The integration of SPAD technology into DSCA provides significant benefits for non-invasive diagnostics and clinical monitoring in neurology and critical care. SPAD sensors offer single-photon sensitivity, high photon detection efficiency, and ultra-short exposure times, enabling fast and accurate tracking of microvascular blood flow dynamics without the need for exogenous contrast agents. These capabilities are particularly valuable for monitoring cerebral perfusion and detecting transient ischemic or hypoperfusion events in a fully non-invasive manner. Our study demonstrates that large-format SPAD-based DSCA can capture neural activity driven blood flow changes with high temporal and spatial resolution, highlighting its potential as a bedside tool for real-time brain function assessment and critical patient monitoring.

## Figures and Tables

**Table 1:** Representative existing DSCA/SCOS system.

Laser	Wavelength (nm)	Applications	Sampling Rate (Hz)	Fiber-Based/Fiberless	Source-Detector Separation (mm)	System Name	Year	Sensor	Ref.
CW	785	Forearm	30	Fiber-based	24	DSCA	2013	EMCCD	[16]
CW	785	Forearm and palm	1	Fiber-based	15	tDSCA	2013	CCD	[25]
CW	785	Forearm	N.A.	Fiberless	30	SCOS	2014	CCD	[19]
CW	671	Phantom	N.A.	Fiberless	18	DSCA	2017	CCD	[31]
CW	785	Forearm and forehead	N.A.	Fibreless	20	SCOS	2018	SPAD (32 × 2)	[18]
CW	785	Forearm, and forehead	300	Fiber-based	25	DSCA	2020	CCD	[32]
CW	785	Forehead	N.A.	Fiber-based	26	SCOS	2023	sCMOS	[33]
VHG holographic	852	Forearm, forehead and arithmetic tests	N.A.	Fiber-based	45	SCOS	2023	sCMOS	[24]
CW	785	Forehead	80	Compact and fiberless	50	SCOS	2024	Sony IMX392	[34]
CW	785	Diabetic	330	Fiber-based	12	DSCA	2024	CCD	[35]
CW	785	Forearm	N.A.	Fiber-based	25	DSCA	2024	Generic photodiode	[36]
CW	808	Wrist, exercise	390	Fiber-based	4.5	SCOS	2024	Basler boost	[37]
CW	785	Forearm, forehead and arithmetic tests	>800	Fiber-based	25	DSCA	2025	SPAD (512 × 512)	ours

**Table 2:** Relationship between TINT_TBIN_ITERATIONS, exposure time, frame readout time, frame time, and frame rate under clock frequency = 20 MHz, TBIN_CLK_PERIODS = 32, and global shutter mode.

TINT_TBIN_ITERATIONS	Exposure Time (s)	Frame Readout Time (s)	Frame Time (s)	Frame Rate (fps)
32	5.1200 × 10^−5^	1.1264 × 10^−3^	1.1776 × 10^−3^	849.2
64	1.0240 × 10^−4^	1.1264 × 10^−3^	1.2288 × 10^−3^	813.8
128	2.0480 × 10^−4^	1.1264 × 10^−3^	1.3312 × 10^−3^	751.2
256	4.0960 × 10^−4^	1.1264 × 10^−3^	1.5360 × 10^−3^	651.0
512	8.1920 × 10^−4^	1.1264 × 10^−3^	1.9456 × 10^−3^	514.0
1024	1.6384 × 10^−3^	1.1264 × 10^−3^	2.7648 × 10^−3^	361.7
2048	3.2768 × 10^−3^	1.1264 × 10^−3^	4.4032 × 10^−3^	227.1
4096	6.5536 × 10^−3^	1.1264 × 10^−3^	7.6800 × 10^−3^	130.2

**Table 3:** SNR values, dark level count, and mean intensity counts of the raw data from a representative subject.

TINT_TBIN_ITERATIONS	SNR	Dark Count Rate (kcps)	Intensity Counts (μ±σ)	ρ (mm)
4096	61.88		3829.70 ± 513.76	10
	20.35	0.28	414.15 ± 22.60	20
	6.8		46.19 ± 4.71	30

## Data Availability

Data availability All data included in this study are available upon request by contact with the corresponding author.

## Appendix A

Substituting $g_{1}\left(\rho ,\tau \right)=G_{1}(\rho ,\tau )/G_{1}(\rho ,0)$ into Equation (2), the speckle contrast expression can be reformulated as

(A1) $$\kappa^{2}\left(\rho ,T\right)=\frac{8\beta }{{\left(FT\right)}^{2}G_{0}^{2}}\sum_{i=1}^{2}\sum_{j=1}^{2}\frac{{\left(-1\right)}^{i+j}}{r_{i}r_{j}{\left(r_{i}+r_{j}\right)}^{4}}\times [X_{ij}\left(T\right)-X_{ij}\left(0\right)+Y_{ij}FT],$$

where $X_{ij}\left(T\right)=[{\left(r_{i}+r_{j}\right)}^{2}\left(K_{0}^{2}+FT\right)+3\left(r_{i}+r_{j}\right)\sqrt{K_{0}^{2}+FT}+3]\times e^{-(r_{i}+r_{j})\sqrt{K_{0}^{2}+FT}}$, $Y_{ij}=\frac{1}{2}{\left(r_{i}+r_{j}\right)}^{2}[1+K_{0}(r_{i}+r_{j})]\times e^{-K_{0}(r_{i}+r_{j})}$. For full details of the derivation, please refer to Ref. [31]. Equation (A1) is a general formular describing the behavior of κ with respect to the exposure time T and ρ. In our theoretical simulation, this equation is used to calculate the corresponding speckle contrast.
